# Draft Genome Sequence of Bacillus cereus Strain UAEU-H3K6M1, a Bacterium with Potential Bioremediation Abilities, Isolated from Petroleum Sludge

**DOI:** 10.1128/MRA.00856-18

**Published:** 2018-07-26

**Authors:** Manal A. Al Hefeiti, Joseph Mafofo, Gess Thoms Xavier, Sathishkumar Ramaswamy, Bincy Baby, Divinlal Harilal, Ranjit Vijayan, S. Salman Ashraf

**Affiliations:** a Department of Chemistry, College of Science, United Arab Emirates University, Al Ain, Abu Dhabi, United Arab Emirates; b Agiomix FZ-LLC, Dubai Science Park, Dubai, United Arab Emirates; c Department of Biology, College of Science, United Arab Emirates University, Al Ain, Abu Dhabi, United Arab Emirates; Louisiana State University

## Abstract

Here, we report the draft genome sequence of Bacillus cereus strain UAEU-H3K6M1, which was isolated from petroleum sludge in the desert. It is composed of around 5.4 Mbp and has a GC content of 35%. Functional annotation and pathway modeling showed multiple genes with potential bioremediation abilities.

## Abstract (precis)

Here, we report the draft genome sequence of Bacillus cereus strain UAEU-H3K6M1, which was isolated from petroleum sludge in the desert. It is composed of around 5.4 Mbp and has a GC content of 35%.

## ANNOUNCEMENT

Bacillus cereus strain UAEU-H3K6M1 was isolated from a petroleum sludge sample and shows interesting bioremediation potential. *In vitro* studies show potential uses with various classes of organic pollutants, including aromatic dyes and contaminants of emerging concern. Here, we report the annotated draft genome sequence (∼5.4 Mbp) of this strain.

Due to ever-increasing pollution of our environment, there is a pressing need to find novel and efficient approaches for remediation of our natural resources, especially water bodies. A number of microbial species that can degrade a range of pollutants have been previously reported (1). We were interested in screening the microbial population in a sample of petroleum sludge from the United Arab Emirates (UAE) to isolate bacteria capable of degrading various classes of aromatic pollutants. A total of 12 different bacterial isolates that showed very interesting bioremediation capacities were purified through conventional culture techniques. The crucial criteria for selection included the efficiency of the isolate in degrading azo dyes under both aerobic and anaerobic conditions. The most promising of these isolates were further screened for their capacity to degrade various classes of contaminants of emerging concern, such as sulfamethoxazole, prometryn, and fluometuron, under aerobic conditions.

Strain UAEU-H3K6M1 was then selected for genome analysis based on the criteria described above. An overnight culture (37°C with gentle shaking) of this strain in nutrient broth was used to isolate genomic DNA using an ABIOPure DNA isolation kit (Alliance Bio, USA). Genomic DNA (1 ng) was used to generate Illumina sequencing libraries using the Nextera XT DNA library prep kit (Illumina) and subsequently sequenced using an Illumina MiSeq instrument. A total of 35,124,272 paired-end reads were generated, which represented a coverage of 242× (total bases/reference genome size = 1,314,502,756/5,427,083 = 242.11). These data were preprocessed for quality using Trimmomatic (version 0.36) (2) and FASTX-Toolkit (version 0.0.13) (3) to remove Illumina adapter sequences and filter out low-quality bases, respectively. Preprocessing reduced the total number of sequence reads to 18,225,662 paired-end reads.

These sequence data were then assembled *de novo* using ABySS (4), IDBA-UD (5), SPAdes (6), and Velvet (7), and an integrated assembly was produced with CISA (8) by using the Tychus pipeline (https://github.com/Abdo-Lab/Tychus). The integrated assembly produced a total of 16 contigs with an *N*_50_ length of 573,109 bp and an *L*_50_ of 4. The largest contig was 1,120,625 bp long. The total assembly sequence was shown to be 5,408,131 bp long with a GC content of 35%. Gene prediction and annotation performed using the NCBI Prokaryotic Genome Annotation Pipeline (PGAP) (https://www.ncbi.nlm.nih.gov/genome/annotation_prok/) identified 5,493 protein-coding genes, 246 pseudogenes, and 40 RNA genes. The most common subsystems identified by rapid genome annotations using RAST (9) were those related to amino acids and derivatives (n = 495 coding sequences [CDSs]), carbohydrates (n = 421), cofactors, vitamins, prosthetic groups, and pigments (n = 223), cell wall and capsule (n = 169), RNA metabolism (n = 149), membrane transport (n = 147), fatty acids, lipids, and isoprenoids (n = 140), protein metabolism (n = 109), nucleosides and nucleotides (n = 107), and defense (n = 105). We identified several protein-coding genes, including copper oxidase and azoreductase 2 and 4, which have potential bioremediation abilities (10).

### Data availability.

The Bacillus cereus strain UAEU-H3K6M1 genome sequence was deposited at DDBJ/ENA/GenBank under the accession number PHQW00000000. The version described in this paper is version PHQW02000000.
