# Correction for Al Hefeiti et al., “Draft Genome Sequence of Bacillus cereus Strain UAEU-H3K6M1, a Bacterium with Potential Bioremediation Abilities, Isolated from Petroleum Sludge”

**DOI:** 10.1128/MRA.01147-18

**Published:** 2018-09-13

**Authors:** Manal A. Al Hefeiti, Joseph Mafofo, Gess Thoms Xavier, Sathishkumar Ramaswamy, Bincy Baby, Divinlal Harilal, Ranjit Vijayan, S. Salman Ashraf

**Affiliations:** a Department of Chemistry, College of Science, United Arab Emirates University, Al Ain, Abu Dhabi, United Arab Emirates; b Agiomix FZ-LLC, Dubai Science Park, Dubai, United Arab Emirates; c Department of Biology, College of Science, United Arab Emirates University, Al Ain, Abu Dhabi, United Arab Emirates

## AUTHOR CORRECTION

Volume 7, no. 3, https://doi.org/10.1128/MRA.00856-18. Page 1: The article byline and author affiliations should appear as given in this correction.

